# Screening and identification of immune-related genes for immunotherapy and prognostic assessment in colorectal cancer patients

**DOI:** 10.1186/s12920-022-01329-2

**Published:** 2022-08-08

**Authors:** Shuwei Wang, Liang Cheng, Fa Jing, Gan Li

**Affiliations:** Department of General Surgery, Wuxi Affiliated Hospital of Nanjing University of Chinese Medicine, Wuxi, 214000 China

**Keywords:** Colorectal cancer, Immune-related gene, Prognostic value, Immunotherapy

## Abstract

**Background:**

Increasing evidence indicates that the immune microenvironment plays a key role in the genesis and progression of colorectal cancer (CRC). This study aimed to establish an immune-related gene (IRG) signature and determine its clinical prognostic value in patients with CRC.

**Methods:**

The RNA sequencing and associated clinical data of CRC were downloaded from The Cancer Genome Atlas (TCGA) database. We then screened for differentially expressed IRGs by intersecting with IRGs obtained from the Immunology Database and Analysis Portal. Functional enrichment analyses were carried out to determine the potential biological functions and pathways of the IRGs. We also explored the specific molecular mechanisms of the IRGs by constructing regulatory networks. Prognostic IRGs were obtained by LASSO regression analysis, and subsequently, gene models were constructed in the TCGA dataset to confirm the predictive capacity of these IRGs. Finally, we used the TIMER tool to assess the immune properties of prognostic IRGs and correlate them with immune cells.

**Results:**

We identified 409 differentially expressed IRGs in patients with CRC. Kyoto Encyclopaedia of Genes and Genomes and Gene Ontology enrichment analyses suggested that these differentially expressed IRGs were significantly related to 102 cancer signalling pathways and various biological functions. Based on the prediction and interaction results, we obtained 59 TF–IRG, 48 miRNA–IRG, and 214 drug–IRG interaction networks for CRC. Four prognostic genes (POMC, TNFRSF19, FGF2, and SCG2) were developed by integrating 47 survival-related IRGs and 42 characteristic CRC genes. The results of gene model showed that patients in the low risk group had better survival outcomes compared to those in the high risk group. The expression of POMC, TNFRSF19, FGF2, and SCG2 was significantly correlated with immune cells.

**Conclusion:**

This study identified some valid IRGs, and these findings can provide strong evidence for precision immunotherapy in patients with CRC.

**Supplementary Information:**

The online version contains supplementary material available at 10.1186/s12920-022-01329-2.

## Background

Colorectal cancer (CRC) was the third most commonly diagnosed cancer and the second leading cause of cancer death in 2020, with approximately 1.9 million new cases and 935,000 deaths worldwide, representing approximately one in ten cancer cases and deaths [1]. Although the five-year survival rate for CRC patients has improved with early screening in developed countries, the outcome for patients with advanced CRC remains unsatisfactory, with a median five-year survival rate of only 12.5% in the USA [2]. Therefore, it is necessary to identify specific biomarkers for early diagnosis and potential therapeutic targets in CRC.

Immunotherapy is gradually becoming the standard treatment for cancer and is as important as surgery, radiotherapy, and chemotherapy. Cancer immunotherapy is designed to promote the immune response of tumour-specific T cells [3]. When fully reprogrammed, T cells are considered the most powerful anti-cancer immune cells. Immunotherapy has not only produced unprecedented clinical results in patients with refractory tumours but has also brought long-term clinical remission to patients with diseases that were historically considered incurable [4]. In recent years, the advent of immune checkpoint inhibitors (ICIs), such as anti-PD-1, has opened up a new landscape for cancer immunotherapy. Nevertheless, the use of ICIs in CRC is currently limited to patients with high microsatellite instability and is only 5–10% effective in CRC patients with microsatellite stability (approximately 90%) [5]. Hence, it is necessary to explore reliable immune-related genes (IRGs) as important immune signatures to improve efficacy and predict prognosis in patients with CRC.

With the presentation of large-scale publicly available gene expression databases, researchers have been able to quickly and accurately identify potential biomarkers for tumour surveillance [6]. The Cancer Genome Atlas (TCGA) is a commonly used database that contains a large amount of transcriptome data and can provide many tumour samples. Multiple immune-related prognostic signatures for lung adenocarcinoma [7], hepatocellular carcinoma [8], breast cancer [9], and clear cell renal cell carcinoma [10] were established from TCGA.

Our study integrated differentially expressed genes obtained from TCGA with IRGs collected from the Immunology Database and Analysis Portal (IMMPort) and conducted an in-depth mining analysis of CRC data. We then analysed and processed the IRGs further by using functional enrichment analyses and regulatory network construction. In addition, we discovered new immune biomarkers associated with CRC prognosis applying LASSO regression analysis. We hope that these findings will lead to accurate prognostic assessment and effective immunotherapy strategies for patients with CRC.

## Methods

### Original data acquisition

The RNA-sequencing and miRNA data were downloaded from TCGA using the UCSC Xena browser (https://xenabrowser.net/datapages/) [11]. The corresponding clinical data for CRC included 353 samples (342 tumour samples and 11 normal samples). The counts per million values were obtained by transforming the original data.

We downloaded the CRC data set numbered GSE39582 from the National Center for Biotechnology Information GEO (Gene Expression Omnibus) (https://www.ncbi.nlm.nih.gov/) database [12]. The data set was processed by the original author and standardized probe expression matrix was downloaded. Meanwhile, the probe annotation information of corresponding platform was downloaded. Convert the probe to gene symbol and eliminate the probe that is not compared to gene symbol. For multiple probes mapped to the same gene symbol, the average value of probes was taken as the expression level of the gene. Then the expression values of four genes FGF2, POMC, SCG2, and TNFRSF19 were selected for subsequent analysis.

### Differential immune-related genes and miRNA screening

The samples were divided into tumour and normal groups. The TMM algorithm in the R (Version 4.1.1) software package edgeR (Version 3.36.0) [13] was used to standardise the raw count and transform it into counts per million, which was used for subsequent analysis. The significance of differences in gene expression was calculated using an unpaired *t*-test and corrected by applying the Benjamini–Hochberg (BH) procedure. Threshold | logFC | > 1 and *p*-value < 0.05 were selected as significant differences in miRNAs and genes expression. The IRGs were collected from the IMMPort database [14] (https://www.immport.org/shared/home), and 1793 different IRGs associated with human cancers were screened out (Additional file 6: Table S1). These genes were intersected with 4747 differentially expressed genes to obtain differentially expressed IRGs in CRC.

### Functional enrichment analyses of IRGs

To explore the potential biological functions and pathways of 409 differentially expressed IRGs, the R software package clusterProfiler [15] was applied to conduct the Kyoto Encyclopedia of Genes and Genomes (KEGG) pathway [16–18] and Gene Ontology (GO) [19] enrichment analysis for IRGs. GO has three ontologies: molecular function (MF), cellular component (CC), and biological process (BP). The results with a *p*-value < 0.05 after BH correction were selected as the most significant enrichment results.

### Survival-associated IRG screening

Clinical survival information and gene expression data from patients with CRC were extracted from TCGA. We used the survival R package (version 2.41-3, https://CRAN.R-project.org/view=Survival) to analyse the impact of differentially expressed IRGs on patient survival and prognosis. Subsequently, we plotted the Kaplan–Meier (K–M) curve to compare overall survival for high- and low-risk expressions, and survival-associated IRGs were identified using a log-rank test (*p* < 0.05).

### Construction of transcription factor (TF)-IRG and miRNA-IRG regulatory network

The over-representation analysis enrichment method was applied to predict TF-target enrichment of differentially expressed genes in protein–protein interaction network, using WebGestalt GAST [20] (http://www.webgestalt.org/option.php). The species selected was hsapiens, the enrichment parameter (the minimum number of enrichment genes) was set at 2, and the results of the Top 10 were displayed. The TF-target gene interaction relations were obtained using Cytoscape [21] (version 3.6.0, http://www.cytoscape.org/) to draw TF-IRG regulatory network.

We used the miRWalk [22] tool to predict regulatory miRNAs of differentially expressed IRGs. The miRNAs obtained by our previous differential analysis were screened out from these miRNAs to construct the relationship between differentially expressed miRNAs and IRGs. Finally, the miRNA-IRG regulatory network was mapped using Cytoscape.

### Establishment of drug–gene interaction network

According to the drug prediction database DGIdb (http://www.dgidb.org/) [23], drug–gene interactions of key differential genes regulated by miRNA and TFs were further predicted by the filtering parameter ‘FDA-approved’. We then constructed the drug–gene interaction network based on the prediction results, using Cytoscape software.

### Prognostic characteristic gene screening and model construction

We screened the characteristic genes of CRC using LASSO regression analysis and integrated them with 47 survival-related IRGs to obtain the prognostic characteristic genes of CRC. The lambda value of the LASSO filter was set to 0.004 by iterative calculation. To confirm the predictive capacity of these IRGs, two thirds of the samples (including 219 CRC samples) in the TCGA dataset were randomly selected using the R language for model construction. The model was validated using one third of the samples (including 110 CRC samples).

### Immune evaluation and mutation analysis of prognostic characteristic genes

We used TIMER tools (https://cistrome.shinyapps.io/timer/) [24] to assess the immune characteristics of four prognostic characteristic genes (GRP, TNFRSF19, FGF2, and SCG2) in order to determine their relevance to immune cells. Simultaneously, the mutation data of four prognostic characteristic genes were downloaded from TCGA genomic data, and the extracted mutation signatures were visualised using R package Maftools (version 2.10.0) [25] .

### Validation of four prognostic characteristic genes from GEO database

To verify the differential expression levels of these four genes (FGF2, POMC, SCG2, and TNFRSF19), we first selected 17 samples with paired paracancer and cancer tissues. The box diagram of the expression of the four genes between the cancer tissue and the paired paracancer tissue samples was then drawn. Paired T test was used to calculate the significance. To verify that these four genes are indeed significantly correlated with prognosis, 550 samples with survival time greater than 30 days were selected first. K–M curve was used to evaluate the association between different gene expression levels and survival prognosis. Expression level higher than or equal to cutoff value is high sample group, expression level lower than cutoff value is low sample group. The cutoff value is judged by the optimal critical value according to the expression value, survival time, and survival state of each gene using R package SurvMiner (Version 0.4.3). To verify the significant association between the four genes and immune cells, we used the Timer algorithm and the Immunedeconv package (version 2.0.0) based on R language [26]. The infiltration levels of macrophages, neutrophils, dendritic cells (DCs), CD8+ T cells, CD4+ T cells and B cells were calculated. Furthermore, spearman correlation and significant P values between the expression level of 4 genes and the level of cell invasion were calculated by corresponding relationship of cancer tissue samples.

## Results

### Confirmation of differentially expressed IRGs and miRNAs

**Fig. 1:**
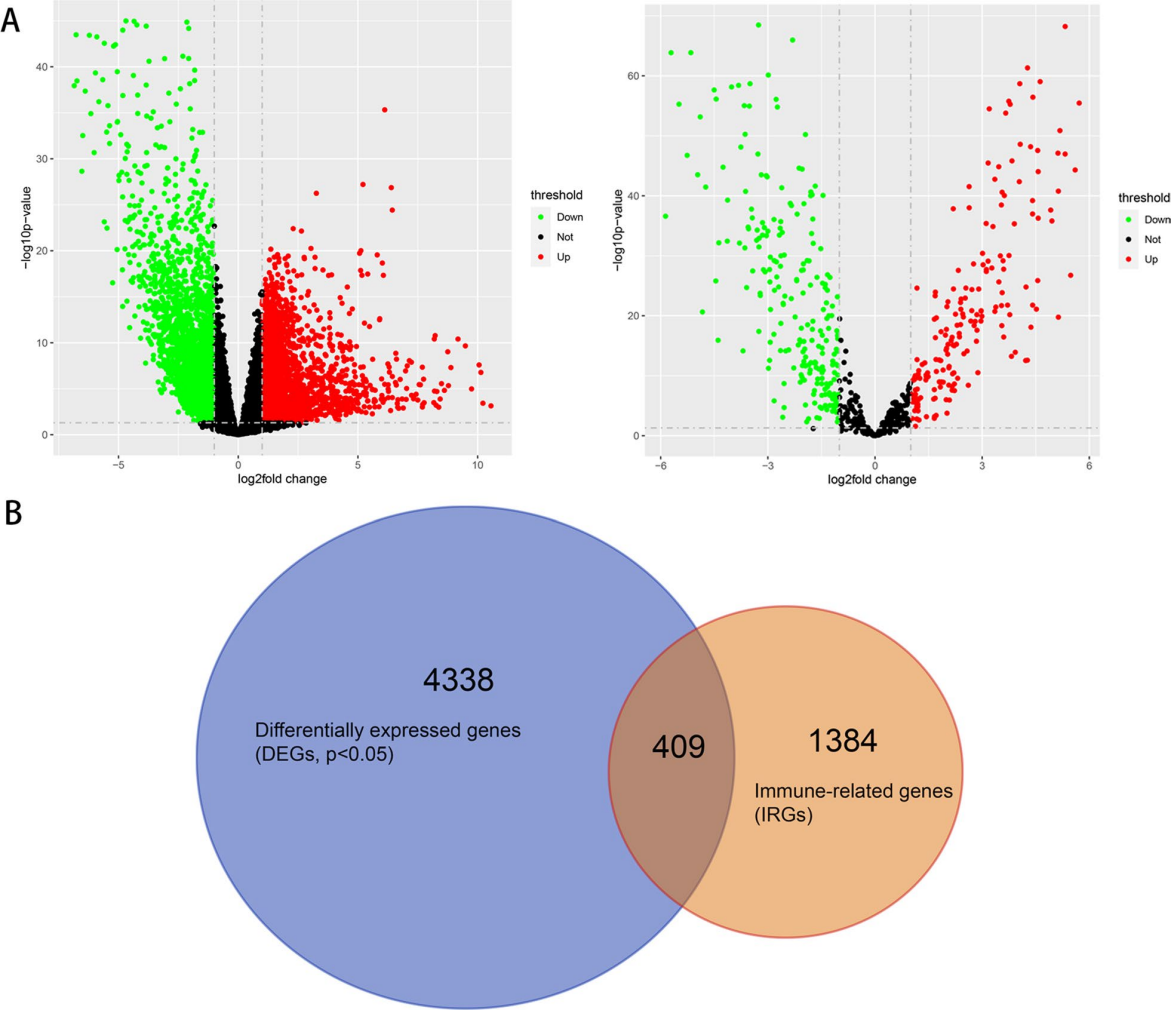
Identification of differentially expressed IRGs and miRNAs. **A** The volcano diagram shows up-regulated genes in red, down-regulated genes in green, and no differentially expressed genes in black. The mRNA volcano on the left and miRNA volcano on the right. **B** Venn diagram, intersection of 4747 differentially expressed genes and 1793 IRGs

A total of 4747 differentially expressed genes, including 2490 up-regulated and 2257 down-regulated genes in CRC, were collected by the above screening method. Meanwhile, 426 differential miRNAs were obtained, of which 193 miRNAs were up-regulated and 233 miRNAs were down-regulated in CRC. In the volcano diagram (Fig. 1A), there is a significant difference between the experimental group and the control group (*p* < 0.05). Then, after intersection of 1793 IRGs downloaded from IMMPort with 4,747 differentially expressed genes, 409 differentially expressed IRGs in CRC were obtained (Fig. 1B).

### Enrichment results of genes

**Fig. 2:**
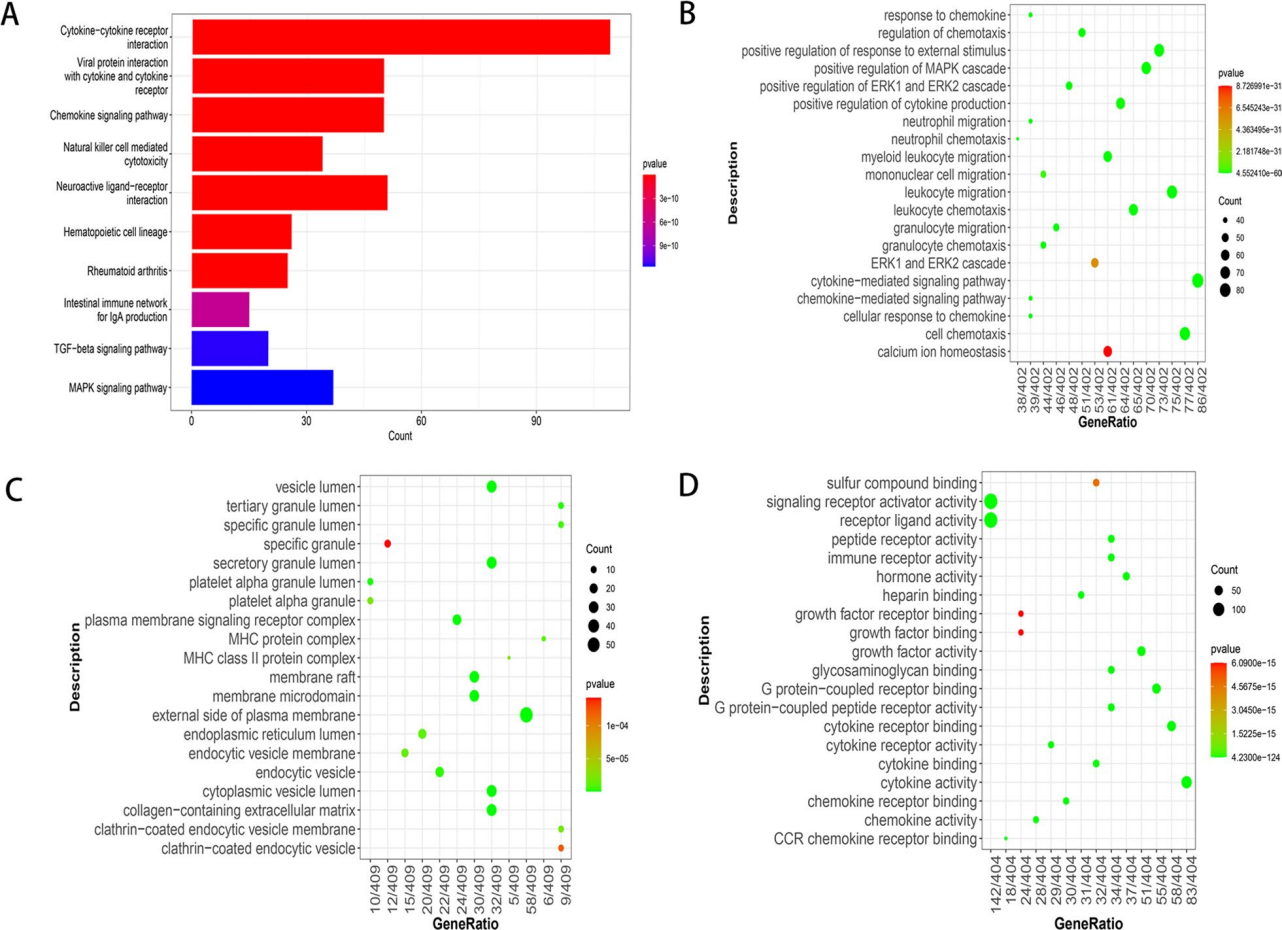
Enrichment results of differentially expressed IRGs. **A** Results of the KEGG pathway enrichment analysis. **B** Results of the GO enrichment analysis in the BP. In the Go-BP bubble diagram, the more red the color is, the smaller the P value is and the more significant the Go-BP is. The larger the bubble, the more genes it contains. **C** Results of the GO enrichment analysis in the CC. **D** Results of the GO enrichment analysis in the MF

The results showed that these 409 differentially expressed IRGs were significantly enriched in 102 KEGG pathways. As shown in Fig. 2A, the top five enriched pathways were cytokine–cytokine receptor interaction, viral protein interaction with cytokine and cytokine receptor, chemokine signalling pathway, natural killer cell-mediated cytotoxicity, and neuroactive ligand-receptor interaction. Moreover, the GO enrichment analysis showed that ‘cell chemotaxis’, ‘external side of plasma membrane’, and ‘receptor ligand activity’ were the most enriched terms in the BP, CC, and MF, respectively (Fig. 2B–D).

### Validation of 47 survival-associated IRGs

**Fig. 3:**
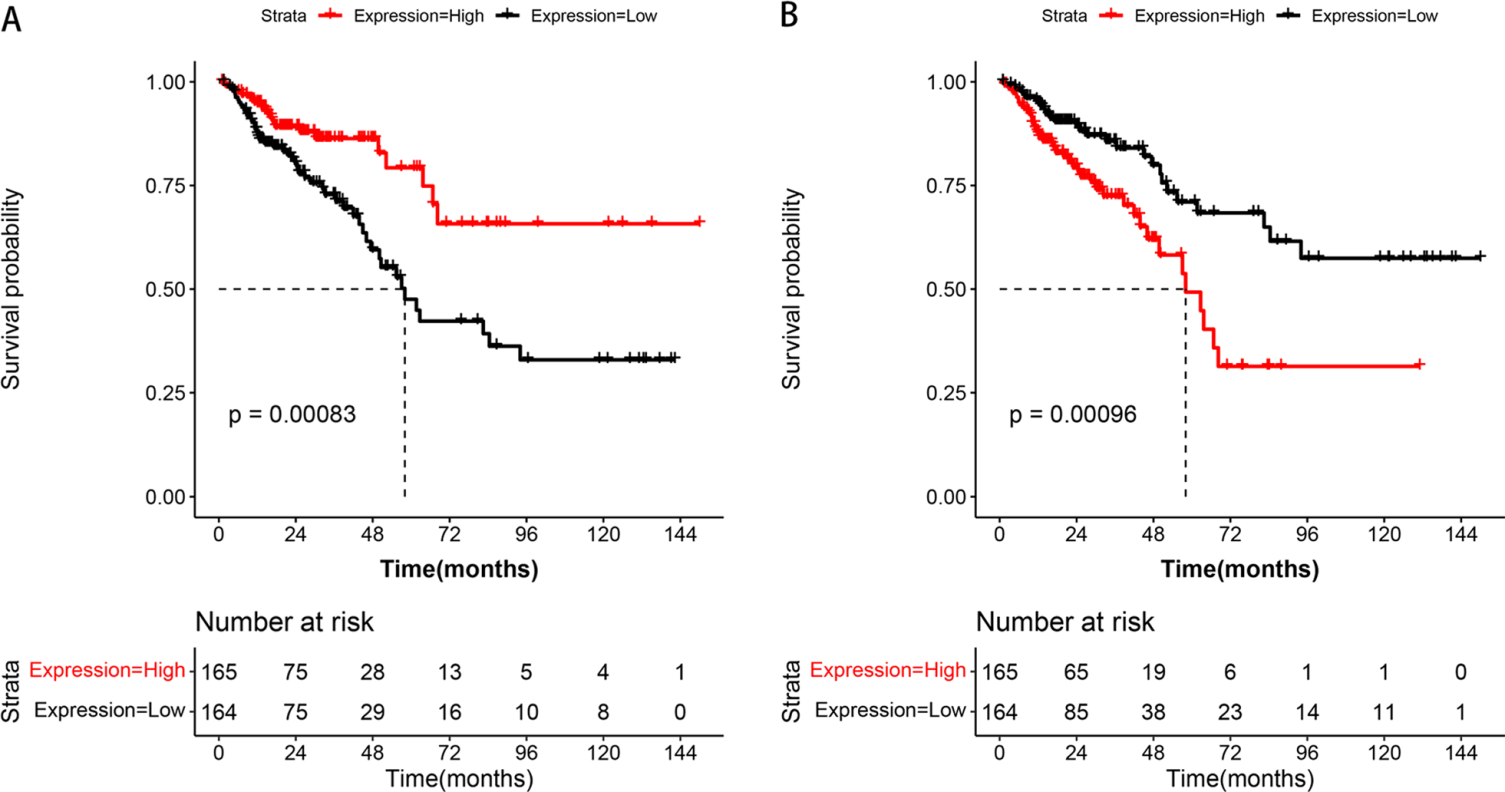
Validation of IRGs associated with survival. **A** Expression level of NRG1 is positively correlated with overall survival. **B** Expression level of PGR is negatively correlated with survival time. The horizontal axis is survival time, the vertical axis is overall survival, red represents the high gene expression group, black represents the low gene expression group

Through survival analysis, we obtained 47 IRGs that were significantly associated with survival, of which 18 were positively correlated and 29 were negatively correlated (Table 1). The K–M survival curve also confirmed the survival difference between the high (n = 165) and low (n = 164) expressing populations. As shown in Fig. 3A, the median survival time of the NRG1 high expression group was significantly longer than that of the low expression group (*p* < 0.05). However, the PGR high expression group showed reduced median survival time (Fig. 3B; *p* < 0.05).

**Table 1:** Validation of 47 survival associated IRGs in CRC

Name	*p* value	Positive/negative correlation	Name	*p* value	Positive/negative correlation
GRP	0.028779	Negative	MC1R	0.029444	Negative
HAMP	0.012122	Negative	FABP2	0.041115	Positive
SEMA6D	0.046234	Positive	MAPT	0.004834	Negative
OLR1	0.023140	Negative	HSPA6	0.013658	Negative
BMP5	0.039142	Positive	GREM1	0.009534	Negative
CCL28	0.034722	Negative	SPP1	0.007463	Negative
CCL21	0.032073	Negative	XCR1	0.029781	Positive
MTNR1A	0.012019	Positive	CCL15	0.024537	Positive
LGR4	0.046138	Negative	ANGPTL1	0.025800	Negative
VIPR1	0.048028	Negative	TNFRSF10C	0.028622	Positive
RBP2	0.046735	Positive	POMC	0.004394	Negative
IL13RA2	0.021061	Positive	UCN3	0.031160	Positive
TLR3	0.039778	Negative	F2RL1	0.013359	Positive
TNFRSF19	0.005499	Negative	IL20RA	0.045089	Negative
CHP2	0.038356	Positive	CD1A	0.017109	Negative
TPM2	0.005409	Negative	INHBB	0.006982	Negative
ULBP3	0.010866	Positive	SLC11A1	0.008031	Negative
SCG2	0.001530	Negative	FGF2	0.018382	Negative
BIRC5	0.047323	Positive	COLEC12	0.037506	Negative
ACVRL1	0.019278	Positive	NTS	0.013250	Positive
PGF	0.046785	Negative	CST4	0.008392	Negative
PGR	0.000961	Negative	AGTR1	0.046148	Negative
NRG1	0.000834	Positive	PTH1R	0.046433	Positive
TGFB2	0.016706	Negative

### Survival analysis adjusting for age and tumor stage

We conducted survival analysis adjusting for age and tumor stage at diagnosis via K-M survival curve. First, our results showed significant differences between patients over 60 years of age in the high-low risk group. Although the results were not significant in patients under 60 years of age, the prognosis of the high-risk group was worse than that of the low-risk group (Additional file 4: Fig. S4A, B). Next, there was a significant difference between the high and low risk groups in stage III-IV patients. No significant results were seen in stage I-II patients, but the prognosis of the high-risk group was worse than that of the low-risk group (Additional file 4: Fig. S4C, D).

### TF-IRG and miRNA-IRG regulatory networks in CRC

**Fig. 4:**
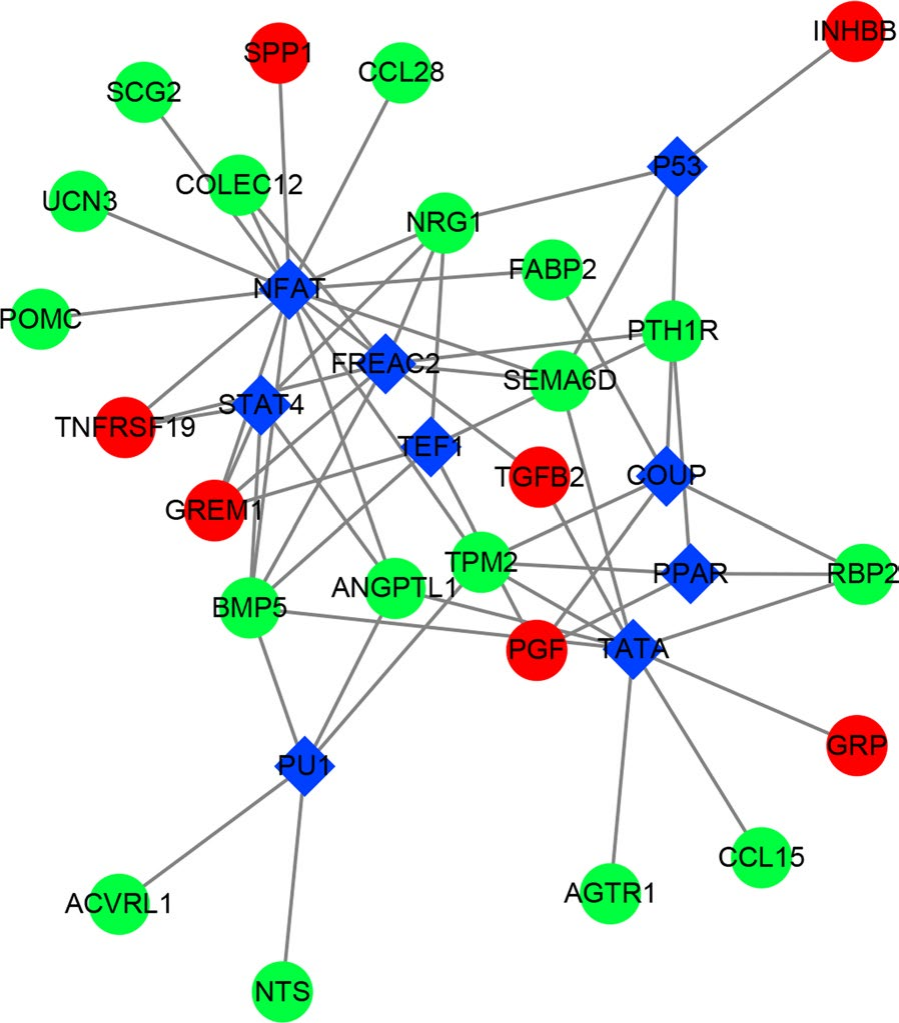
TF-IRG regulatory network in CRC. The circle represents IRG, red is up-regulated gene, green is down-regulated gene; the blue diamond represents TF

According to the prediction results of the over-representation analysis enrichment method, we obtained 59 pairs of TF-IRG interactions, including 9 TFs (NFAT, COUP, STAT4, TEF1, P53, PPAR, TATA, FREAC2, PU1) and 24 IRGs, of which 7 IRGs (TNFRSF19, TGFB2, GREM1, SPP1, PGF, INHBB, and GRP) were upregulated and 17 IRGs (SEMA6D, BMP5, TPM2, SCG2, NRG1, FABP2, ANGPTL1, POMC, UCN3, COLEC12, RBP2, PTH1R, CCL15, AGTR1, ACVRL1, NTS, and CCL28) were downregulated in CRC. Based on the above results, a complex TF-IRG network diagram for CRC was constructed using Cytoscape (Fig. 4).

**Fig. 5:**
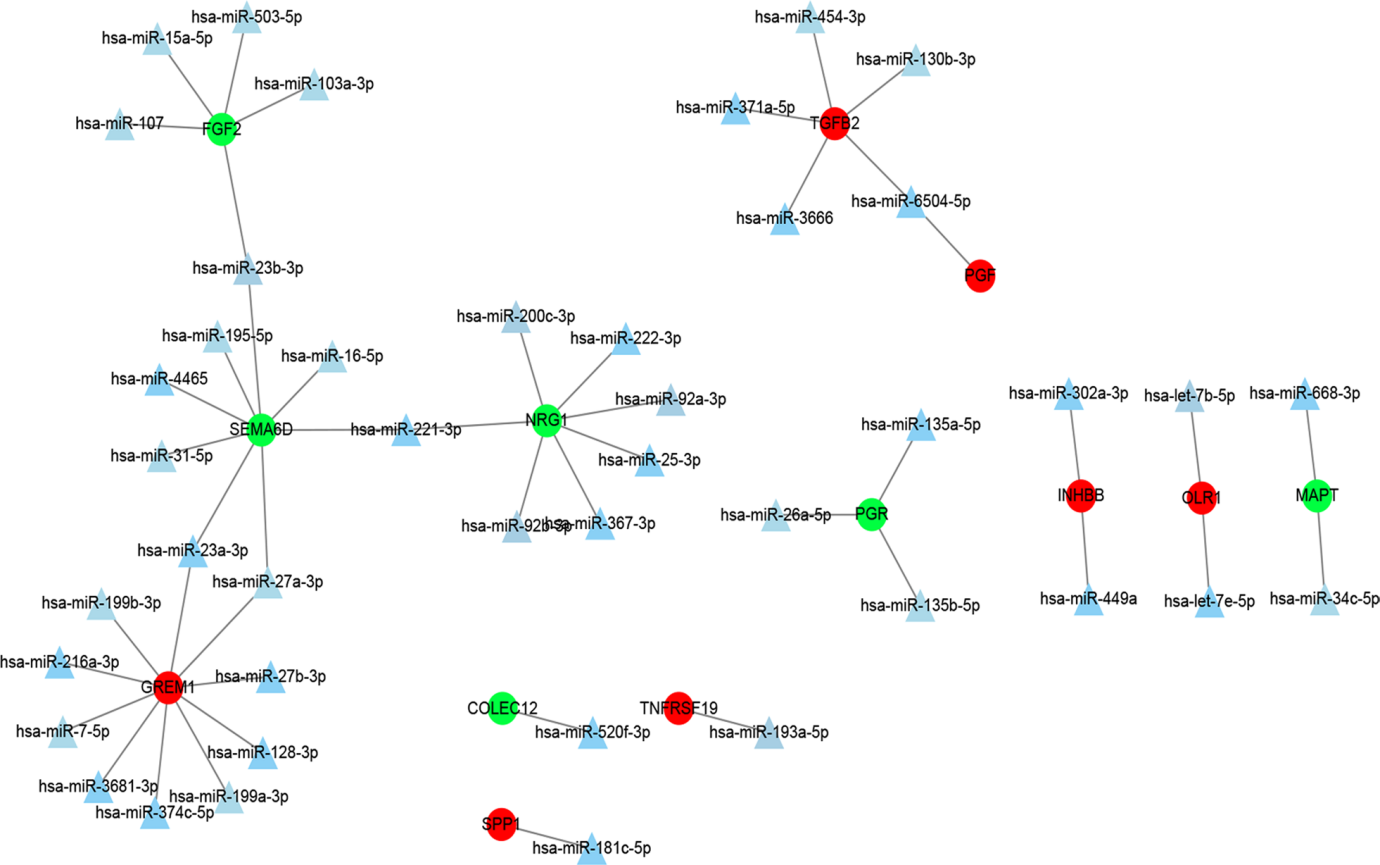
MiRNA-IRG regulatory network in CRC. The triangle represents miRNA; Red circles represent up-regulated genes, green circles represent down-regulated genes

After integrating the 426 differential miRNAs previously obtained and the targeted miRNAs of the predicted IRGs using the miRWalk tool, we identified 43 miRNAs, 13 IRGs, and 48 miRNA-IRG relationship pairs, as shown in Fig. 5.

### Drug–gene interaction network in CRC

**Fig. 6:**
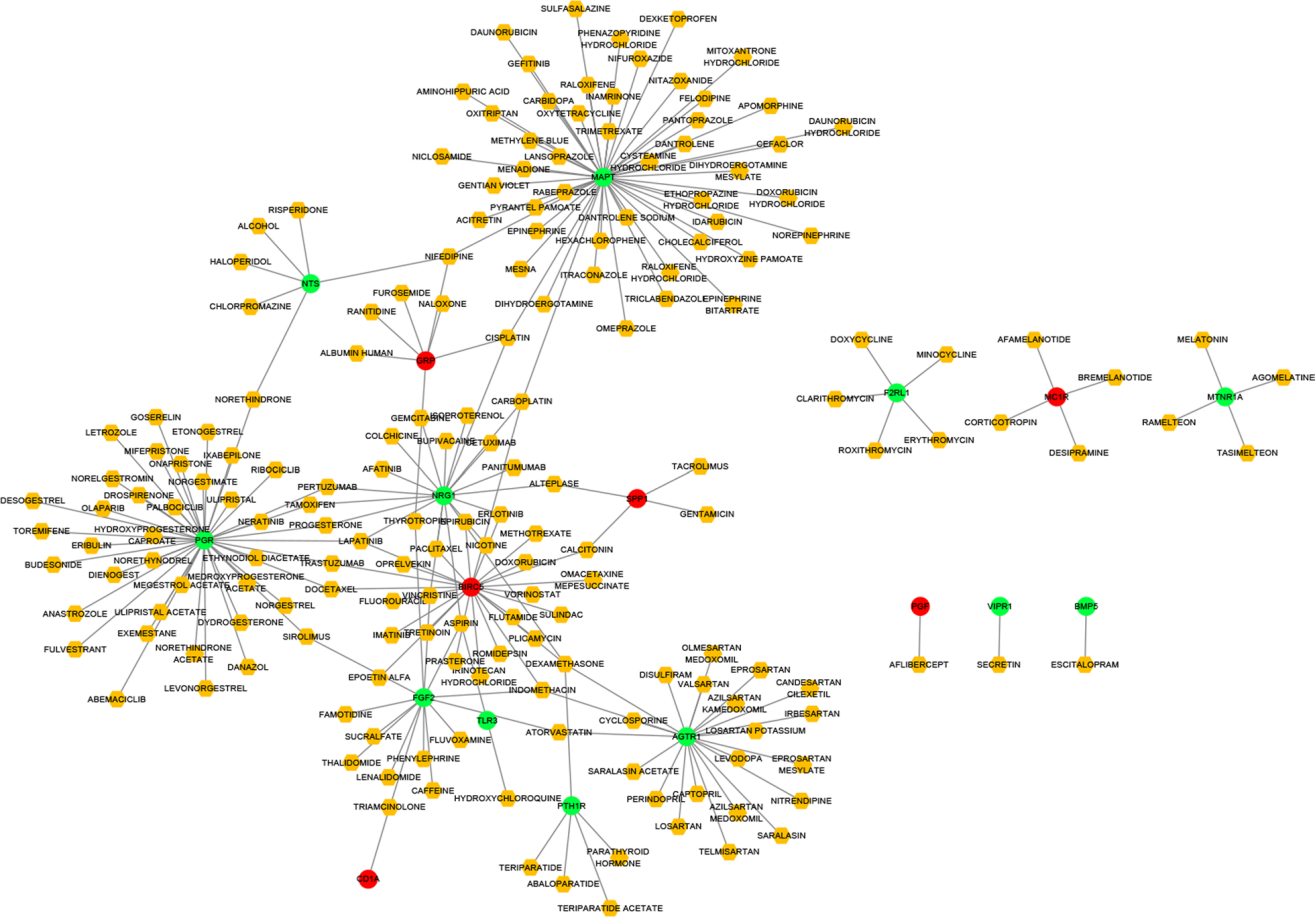
Drug-IRG interaction network in CRC. The yellow hexagon represents small drug molecules; the red circles represent up-regulated genes and the green circles represent down-regulated genes

Based on the drug–gene interaction information of TF- and miRNA-regulated IRGs in the DGIdb database, we obtained 214 relationship pairs between small drug molecules and IRGs, including 18 IRGs (MAPT, NST, PGR, NRG1, FGF2, TLR3, PTH1R, AGTR1, F2RL1, MTNR1A, VIPR1, BMP5, GRP, BIRC5, SPP1, CD1A, PGF, and MC1R) and 195 drugs. The drug–gene interaction network diagram was plotted using Cytoscape (Fig. 6).

### Screening and modelling of disease characteristic genes

**Fig. 7:**
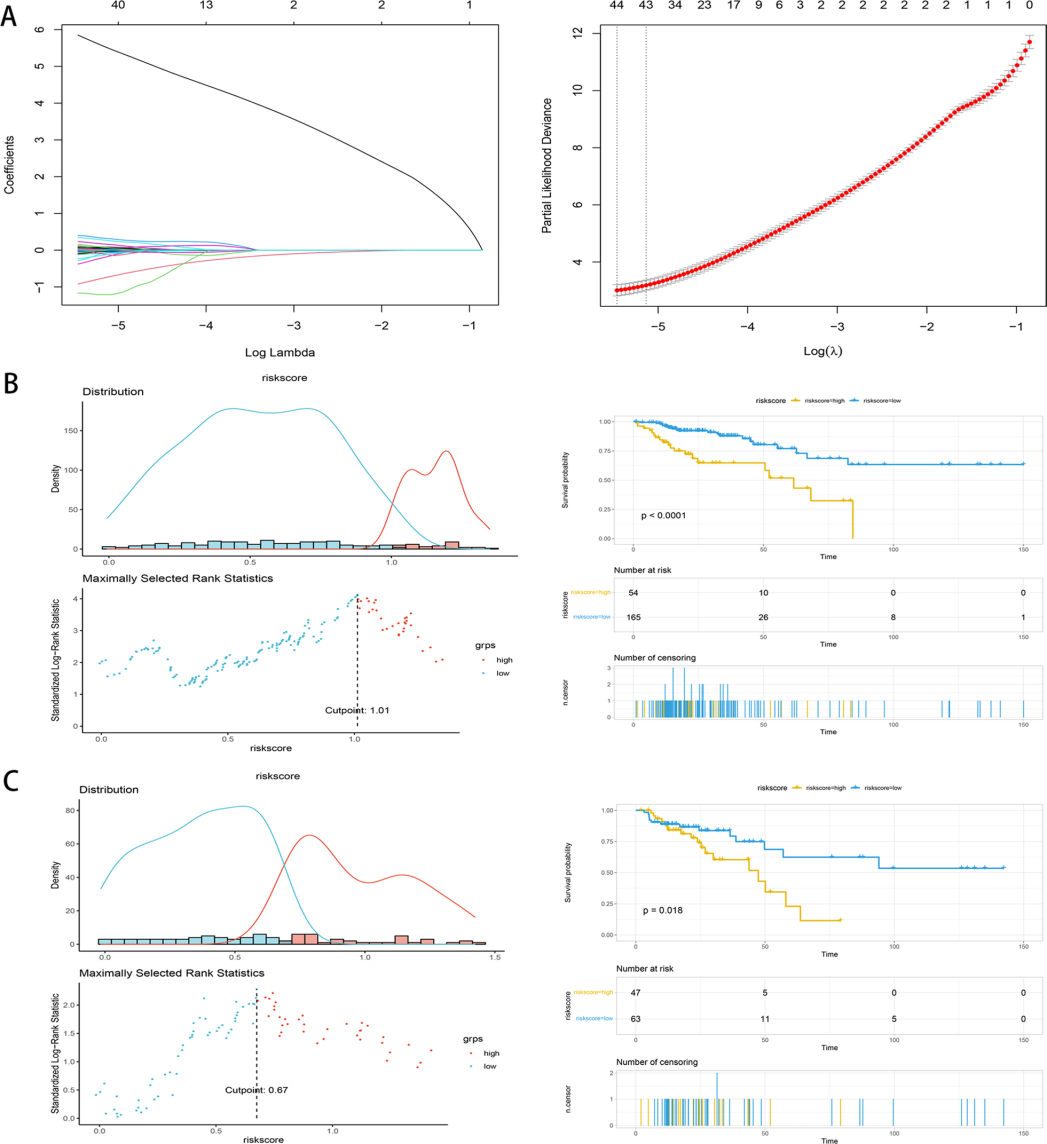
Screening and modeling of prognostic characteristic IRGs in CRC. **A** The characteristic genes were screened by LASSO method. **B** IRGs model was constructed in TCGA dataset. **C** Validation diagram of gene model

By integrating gene node information in multiple networks, 42 CRC characteristic genes were screened using the LASSO method (Fig. 7A), and four prognostic characteristic genes (POMC, TNFRSF19, FGF2, and SCG2) were obtained by further intersection with 47 survival-related IRGs. To further confirm the predictive effect of these IRGs, 329 CRC samples from TCGA were used to construct a gene model. As shown in Fig. 7B, patients in the low-risk group had a better survival prognosis than those in the high-risk group, which was consistent with the model validation results in Fig. 7C.

### Immunocorrelation and mutation analysis of prognostic characteristic genes

**Fig. 8:**
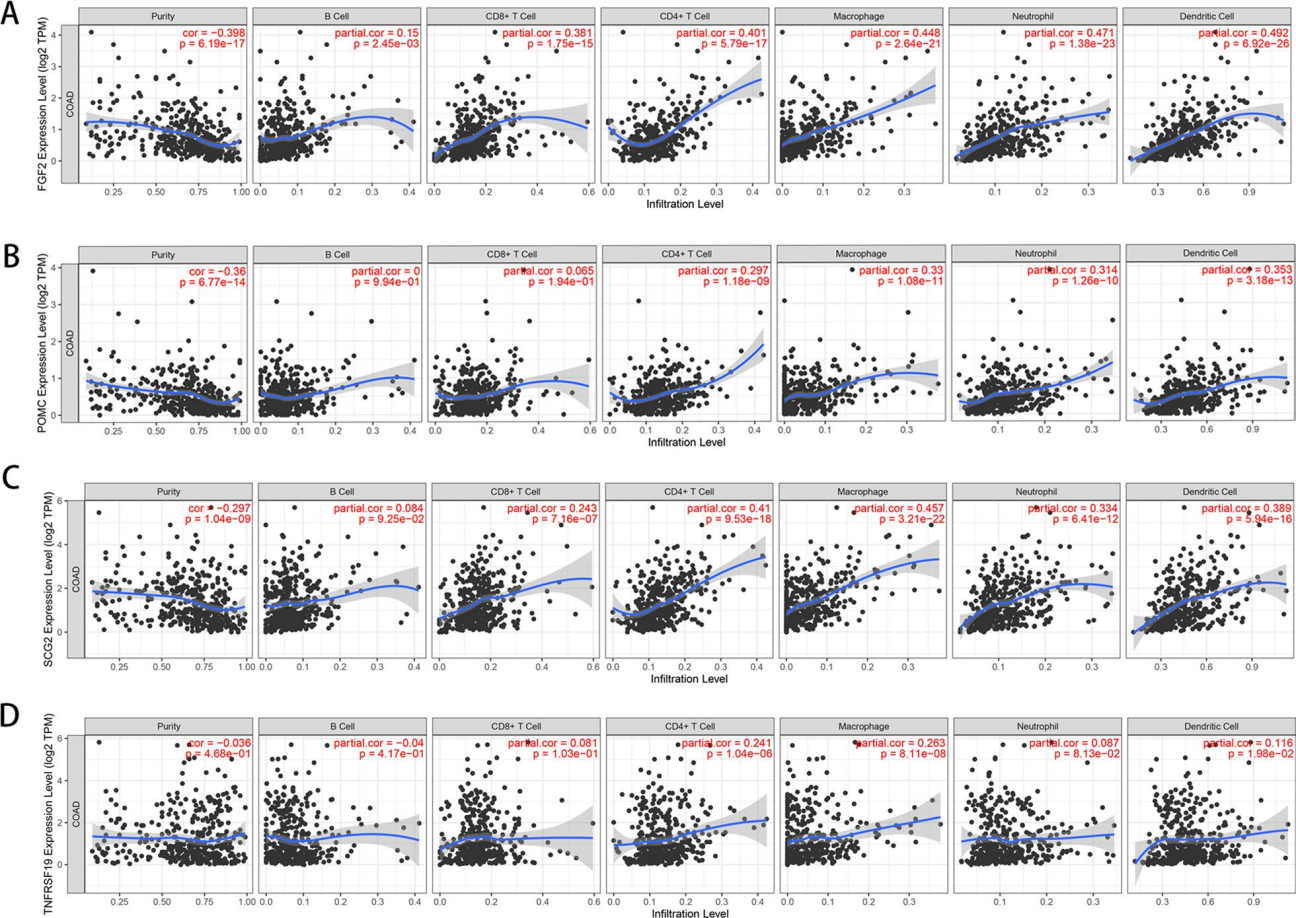
Immunocorrelation of prognostic characteristic IRGs. **A** Immune cell correlation of FGF2. **B** Immune cell correlation of POMC. **C** Immune cell correlation of SCG2. **D** Immune cell correlation of TNFRSF19

**Fig. 9:**
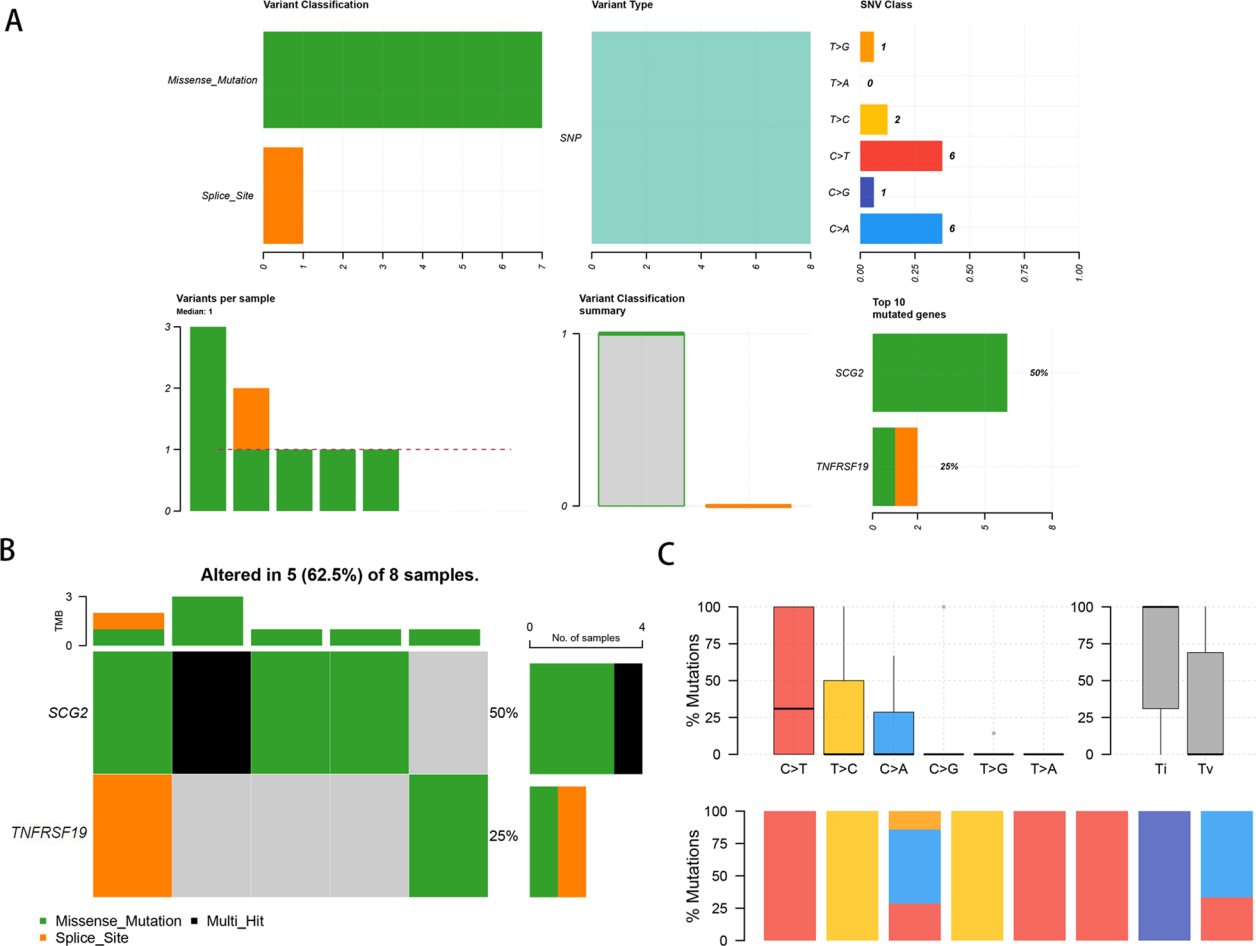
Mutation analysis of prognostic characteristic IRGs. **A** Summary statistical of TNFRSF19 and SCG2 mutation information including “Variant Classification”, “Variant Type”, “SNV Class”, “Variants per Sample”, “Variant Classification Summary” and “Top 10 mutated genes”. **B** Waterfall diagram: mutation statistics in each sample. **C** The overall distribution of six different mutational transformations

Our results showed that four prognostic characteristic genes (FGF2, POMC, SCG2, and TNFRSF19) were significantly related to a variety of immune cells. As shown in Fig. 8, the expression of POMC, TNFRSF19, FGF2, and SCG2 was significantly associated with macrophages, neutrophils, DCs, CD8+ T cells, CD4+ T cells and B cells (*p* < 0.05). Mutation analysis was also performed for the four prognostic characteristic genes, but Figure 9A only shows the summary gene mutation information for TNFRSF19 and SCG2, because there was no gene mutation information for POMC and FGF2 available in TCGA. Due to the small number of genes and mutation sites, the waterfall diagram of the mutation analysis was not obvious (Fig. 9B). Figure 9C displays the overall distribution of six different mutational transformations (C > T, T > C, C > A, C > G, T > G, and T > A).

### Validation results from the GEO database

As shown in Additional file 1: Fig. S1, it can be found that the expression levels of FGF2 and SCG2 in CRC are significantly down-regulated, while the expression levels of POMC and TNFRSF19 are significantly up-regulated, which is consistent with the previous difference results. In addition, it can be seen from Additional file 2: Fig. S2 that all four genes showed worse prognosis after high expression. Except TNFRSF19, the other three genes were significantly correlated with prognosis (*p* < 0.05). Further correlation heat maps showed significant correlations between genes (FGF2 and SCG2) and all six immune cells. POMC was significantly correlated with other immune cells except neutrophils. There was also a significant correlation between TNFRSF19 and macrophages and B cells (Additional file 3: Fig. S3). These conclusions are basically consistent with the previous analysis results.

## Discussion

CRC is currently the second leading cause of cancer-related death, with malignant progression and metastasis leading to high mortality in advanced CRC [1]. Immune components in the tumour microenvironment have recently been reported to influence tumour progression in various cancers, including CRC [27]. As some immune cells are further polarised, the adaptive immune response is reversed, ultimately accelerating cancer cell proliferation, tumour angiogenesis, progression, and metastasis [28]. Therefore, the regulation of the tumour immune microenvironment has become an attractive clinical strategy for cancer treatment. With the launch of the first cancer immunotherapy (recombinant cytokine interferon-α for hairy cell leukaemia) in 1986, more than a dozen immunotherapies have been approved for a variety of cancers, including melanoma, advanced stomach cancer, bladder cancer, hepatocellular carcinoma, prostate cancer, kidney cancer, and non-small cell lung cancer [29]. Unlike chemotherapy, which kills cancer cells directly, cancer immunotherapies attack tumour cells by activating the host's immune system with fewer off-target effects [30]. However, the role of IRGs as important immune signatures in CRC has not yet been fully explored. In this study, we acquired 409 differentially expressed IRGs in CRC from TCGA and IMMPort using the above screening methods. Furthermore, KEGG enrichment analyses indicated that these differentially expressed IRGs were significantly associated with 102 cancer signalling pathways. In patients undergoing colorectal cancer surgery, IRGs related to the enrichment pathway for natural killer cell-mediated cytotoxicity was significantly reduced after primary tumour resection [31]. This also confirms the value of these IRGs in the treatment of CRC. In addition, GO enrichment analysis suggested that these IRGs possess multiple molecular functions and engagement in various biological processes such as cell chemotaxis and receptor ligand activity, which are involved in tumour development and metastasis [32, 33].

Based on the prediction and interaction results, we obtained 59 TF-IRG and 48 miRNA-IRG interaction networks in CRC. TFs such as NFAT have been experimentally confirmed to be involved in the development and progression of CRC [34]. Recent studies have also suggested that the expression of NFATc1 is closely related to the clinical stage and metastasis of CRC, and the application of Ca^2+^–calcineurin–NFAT signalling inhibitors can inhibit CRC metastasis in mouse models [35]. Moreover, another TF that we obtained, P53, not only controls the expression of anticancer genes through transcriptional activity, but also plays a tandem role with various signalling pathways in CRC [36]. The 43 miRNAs obtained also play a variety of roles in cancer genesis, progression, metastasis, and recurrence. For example, high miR-181c expression was significantly associated with recurrence in stage II CRC patients [37]. Furthermore, Hernandez demonstrated that the overexpression of miRNA-26a increased the proliferation and migration rates of CRC cells in vitro [38]. Finally, we constructed 214 drug–IRG regulatory networks based on the drug–gene interaction results of TF- and miRNA-regulated IRGs in CRC. These results provide a strong basis for precision immunotherapy in CRC patients.

The latest global statistics show that the five-year relative survival rate of CRC reached 64% in the United States from 2009 to 2015, was nearly 57% in China from 2012 to 2015, and was less than 50% in many Eastern and Southern European countries [39]. In particular, metastatic CRC has a five-year survival rate of only 14% in Europe, despite advances in treatment [40]. Therefore, early diagnosis and treatment of CRC is highly effective in order to significantly improve the survival rate of patients. In this study, we identified four prognostic genes for CRC (POMC, TNFRSF19, FGF2, and SCG2) by integrating 47 survival-related IRGs and 42 CRC characteristic genes. We believe that these findings may lead to more early diagnostic biomarkers for CRC and improvement of the five-year survival rate of patients. Furthermore, the expression of POMC, TNFRSF19, FGF2, and SCG2 was significantly associated with immune cells, such as macrophages, neutrophils, DCs, CD8+ T cells, CD4+ T cells and B cells. Immune cells in the tumour microenvironment can antagonise or promote tumours. It has been demonstrated that a high proportion of infiltrating dendritic, CD8+ T, and CD4+ T cells leads to better clinical outcomes in CRC patients [41]. However, recent studies suggest that macrophages increase the migration, invasion, and metastatic ability of tumours, reflecting their tumour-promoting effect in CRC [42]. Prognostic genes may therefore serve as targeted entry points for CRC immunotherapy.

We used the information from the group of Li [43] to discuss related analysis of MSI via R software. A total of 342 samples who have MSI information and related gene expression information after matching our information and the data in Li’s database. Then, we conducted statistical analysis via using limma package to compare MSI and MSS. According to the threshold FDR<0.05 and | logFC | > 1, a total of 1294 differentially expressed genes were obtained. Next, we got 160 differentially IRGs after taking the intersection between the above 1294 differentially expressed genes and the IRGs in IMMPort database (https://www.immport.org/shared/home). However, no statistical difference of immune gene expressions was observed between MSI and MSS groups. In order to confirm the conclusion, we conducted an analysis about the levels of infiltration of 22 immune cells in each sample based on gene expression matrix via CIBERSORT [44]. Except for T cell Gamma Delta and Neutrophi, which showed a significant difference in the level of cell infiltration, the other cells showed no significant difference in infiltration between the two groups, which once again proved that there was little difference in immunity between MSI and MSS groups in the samples of this study (Additional file 5: Fig. S5).

## Conclusion

In this study, bioinformatics analysis revealed 59 TF-IRG and 48 miRNA-IRG regulatory networks in CRC, which provides theoretical basis for further improving the biological mechanism of CRC occurrence, development and metastasis. We also identified several valid characteristic survival-related IRGs (POMC, TNFRSF19, FGF2, and SCG2) that could effectively assess the prognosis of patients with CRC. These potential immune biomarkers could be used to develop precise and effective personalised immunotherapy strategies for CRC patients.

## Supplementary Information

**Additional file 1. Fig. S1**: The box diagram of expression of prognostic characteristic IRGs. From A to D, the expression box diagram of FGF2, SCG2, POMC, and TNFRSF19 is shown in the figure. Green is the paracancer tissue, yellow is the cancer tissue, and the line in the middle indicates that they belong to the same sample.

**Additional file 2. Fig. S2**: Correlation between IRGs and prognosis from GEO database. The survival curves of FGF2, SCG2, POMC, and TNFRSF19 were shown from A to D. In the figure, red represents high expression group and black represents low expression group.

**Additional file 3. Fig. S3**: Validation of immunocorrelation of IRGs from GEO database. From left to right are heat maps of correlations between FGF2, POMC, SCG2, TNFRSF19 and immune cells. The top left corner of each small square in the figure represents significance, and * represents p < 0.05, ** represents p < 0.01. The lower right corner shows correlation, green to red shows significance from negative to positive, and the deeper the correlation coefficient is, the greater the absolute value.

**Additional file 4. Fig. S4**: The K-M survival curve of survival analysis adjusting for age and tumor stage. (A) K-M survival curve of patients under 60 years in the high-low risk group. (B) K-M survival curve of patients over 60 years in the high-low risk group. (C) K-M survival curve of patients in stage I-II. (D) K-M survival curve of patients in stage III-IV.

**Additional file 5. Fig. S5**: The levels of immune cells infiltration in MSI and MSS groups. T cell Gamma Delta and Neutrophi showed a significant difference in the level of cell infiltration (p < 0.05), while the other cells showed no significant difference in infiltration between the two groups (p > 0.05).

**Additional file 6. Table S6**: Details of 1793 different IRGs collected from the IMMPort database.

## Data Availability

The datasets used in this study are available from the TCGA database (https://xenabrowser.net/datapages/), GEO database (https://www.ncbi.nlm.nih.gov/), the IMMPort database (https://www.immport.org/shared/home), and the DGIdb database (http://www.dgidb.org/).

## Abbreviations

CRC: Colorectal cancer
IRG: Immune-related gene
TCGA: The cancer genome atlas
ICIs: Immune checkpoint inhibitors
IMMPort: Immunology database and analysis portal
BH: Benjamini–Hochberg
KEGG: Kyoto encyclopedia of genes and genomes
GEO: Gene expression omnibus
GO: Gene ontology
MF: Molecular function
CC: Cellular component
BP: Biological process
K-M: Kaplan–Meier
TF: Transcription factor

## References

[1] Sung H, Ferlay J, Siegel RL, Laversanne M, Soerjomataram I, Jemal A, Bray F (2021). Global cancer statistics 2020: GLOBOCAN estimates of incidence and mortality worldwide for 36 cancers in 185 Countries. CA Cancer J Clin.

[2] Ganesh K, Stadler ZK, Cercek A, Mendelsohn RB, Shia J, Segal NH, Diaz LA (2019). Immunotherapy in colorectal cancer: rationale, challenges and potential. Nat Rev Gastroenterol Hepatol.

[3] Borst J, Ahrends T, Babala N, Melief CJM, Kastenmuller W (2018). CD4(+) T cell help in cancer immunology and immunotherapy. Nat Rev Immunol.

[4] Guedan S, Ruella M, June CH (2019). Emerging cellular therapies for cancer. Annu Rev Immunol.

[5] Liu C, Liu R, Wang B, Lian J, Yao Y, Sun H, Zhang C, Fang L, Guan X, Shi J (2021). Blocking IL-17A enhances tumor response to anti-PD-1 immunotherapy in microsatellite stable colorectal cancer. J Immuno Ther Cancer.

[6] Lin P, Guo YN, Shi L, Li XJ, Yang H, He Y, Li Q, Dang YW, Wei KL, Chen G (2019). Development of a prognostic index based on an immunogenomic landscape analysis of papillary thyroid cancer. Aging.

[7] Sun S, Guo W, Wang Z, Wang X, Zhang G, Zhang H, Li R, Gao Y, Qiu B, Tan F (2020). Development and validation of an immune-related prognostic signature in lung adenocarcinoma. Cancer Med.

[8] Chen W, Ou M, Tang D, Dai Y, Du W (2020). Identification and validation of immune-related gene prognostic signature for hepatocellular carcinoma. J Immunol Res.

[9] Huang Y, Chen L, Tang Z, Min Y, Yu W, Yang G, Zhang L (2021). A novel immune and stroma related prognostic marker for invasive breast cancer in tumor microenvironment: a TCGA based study. Front Endocrinol.

[10] Zhang L, Li J, Zhang M, Wang L, Yang T, Shao Q, Liang X, Ma M, Zhang N, Jing M (2021). Identification of a six-gene prognostic signature characterized by tumor microenvironment immune profiles in clear cell renal cell carcinoma. Front Genet.

[11] Goldman M, Craft B, Swatloski T, Cline M, Morozova O, Diekhans M, Haussler D, Zhu J (2015). The UCSC cancer genomics browser: update 2015. Nucleic Acids Res.

[12] Barrett T, Troup DB, Wilhite SE, Ledoux P, Rudnev D, Evangelista C, Kim IF, Soboleva A, Tomashevsky M, Edgar R (2007). NCBI GEO: mining tens of millions of expression profiles--database and tools update. Nucleic Acids Res.

[13] Robinson MD, McCarthy DJ, Smyth GK (2010). edgeR: a Bioconductor package for differential expression analysis of digital gene expression data. Bioinformatics.

[14] Bhattacharya S, Dunn P, Thomas CG, Smith B, Schaefer H, Chen J, Hu Z, Zalocusky KA, Shankar RD, Shen-Orr SS (2018). ImmPort, toward repurposing of open access immunological assay data for translational and clinical research. Sci Data.

[15] Tianzhi W, Erqiang H, Shuangbin X, Chen M, Guo P, Dai Z, Feng T, Zhou L (2021). clusterProfiler 4.0: a universal enrichment tool for interpreting omics data. Innov.

[16] Kanehisa M, Goto S (2000). KEGG: kyoto encyclopedia of genes and genomes. Nucleic Acids Res.

[17] Kanehisa M (2019). Toward understanding the origin and evolution of cellular organisms. Protein Sci.

[18] Kanehisa M, Furumichi M, Sato Y, Ishiguro-Watanabe M, Tanabe M (2021). KEGG: integrating viruses and cellular organisms. Nucleic Acids Res.

[19] Gene Ontology C (2015). Gene ontology consortium: going forward. Nucleic Acids Res.

[20] Liao Y, Wang J, Jaehnig EJ, Shi Z, Zhang B (2019). WebGestalt 2019: gene set analysis toolkit with revamped UIs and APIs. Nucleic Acids Res.

[21] Otasek D, Morris JH, Boucas J, Pico AR, Demchak B (2019). Cytoscape Automation: empowering workflow-based network analysis. Genome Biol.

[22] Sticht C, De La Torre C, Parveen A, Gretz N (2018). miRWalk: An online resource for prediction of microRNA binding sites. PLoS ONE.

[23] Cotto KC, Wagner AH, Feng YY, Kiwala S, Coffman AC, Spies G, Wollam A, Spies NC, Griffith OL, Griffith M (2018). DGIdb 3.0: a redesign and expansion of the drug–gene interaction database. Nucleic Acids Res.

[24] Li T, Fan J, Wang B, Traugh N, Chen Q, Liu JS, Li B, Liu XS (2017). TIMER: A web server for comprehensive analysis of tumor-infiltrating immune cells. Cancer Res.

[25] Mayakonda A, Lin DC, Assenov Y, Plass C, Koeffler HP (2018). Maftools: efficient and comprehensive analysis of somatic variants in cancer. Genome Res.

[26] Sturm G, Finotello F, List M (2020). Immunedeconv: An R package for unified access to computational methods for estimating immune cell fractions from Bulk RNA-sequencing data. Methods Mol Biol.

[27] Sharma P, Hu-Lieskovan S, Wargo JA, Ribas A (2017). Primary, adaptive, and acquired resistance to cancer immunotherapy. Cell.

[28] Zhang Y, Song J, Zhao Z, Yang M, Chen M, Liu C, Ji J, Zhu D (2020). Single-cell transcriptome analysis reveals tumor immune microenvironment heterogenicity and granulocytes enrichment in colorectal cancer liver metastases. Cancer Lett.

[29] Riley RS, June CH, Langer R, Mitchell MJ (2019). Delivery technologies for cancer immunotherapy. Nat Rev Drug Discov.

[30] Bergman PJ (2019). Cancer immunotherapies. Vet Clin North Am Small Anim Pract.

[31] Niavarani SR, Lawson C, Bakos O, Boudaud M, Batenchuk C, Rouleau S, Tai LH (2019). Lipid accumulation impairs natural killer cell cytotoxicity and tumor control in the postoperative period. BMC Cancer.

[32] Karin N, Razon H (2018). Chemokines beyond chemo-attraction: CXCL10 and its significant role in cancer and autoimmunity. Cytokine.

[33] Du Z, Lovly CM (2018). Mechanisms of receptor tyrosine kinase activation in cancer. Mol Cancer.

[34] Gang W, Yu-Zhu W, Yang Y, Feng S, Xing-Li F, Heng Z (2019). The critical role of calcineurin/NFAT (C/N) pathways and effective antitumor prospect for colorectal cancers. J Cell Biochem.

[35] Shen T, Yue C, Wang X, Wang Z, Wu Y, Zhao C, Chang P, Sun X, Wang W (2021). NFATc1 promotes epithelial-mesenchymal transition and facilitates colorectal cancer metastasis by targeting SNAI1. Exp Cell Res.

[36] Cho YH, Ro EJ, Yoon JS, Mizutani T, Kang DW, Park JC, Il Kim T, Clevers H, Choi KY (2020). 5-FU promotes stemness of colorectal cancer via p53-mediated WNT/beta-catenin pathway activation. Nat Commun.

[37] Yamazaki N, Koga Y, Taniguchi H, Kojima M, Kanemitsu Y, Saito N, Matsumura Y (2017). High expression of miR-181c as a predictive marker of recurrence in stage II colorectal cancer. Oncotarget.

[38] Coronel-Hernandez J, Lopez-Urrutia E, Contreras-Romero C, Delgado-Waldo I, Figueroa-Gonzalez G, Campos-Parra AD, Salgado-Garcia R, Martinez-Gutierrez A, Rodriguez-Morales M, Jacobo-Herrera N (2019). Cell migration and proliferation are regulated by miR-26a in colorectal cancer via the PTEN-AKT axis. Cancer Cell Int.

[39] Li N, Lu B, Luo C, Cai J, Lu M, Zhang Y, Chen H, Dai M (2021). Incidence, mortality, survival, risk factor and screening of colorectal cancer: a comparison among China, Europe, and northern America. Cancer Lett.

[40] Mauri G, Sartore-Bianchi A, Russo AG, Marsoni S, Bardelli A, Siena S (2019). Early-onset colorectal cancer in young individuals. Mol Oncol.

[41] Picard E, Verschoor CP, Ma GW, Pawelec G (2020). Relationships between immune landscapes, genetic subtypes and responses to immunotherapy in colorectal cancer. Front Immunol.

[42] Wei C, Yang C, Wang S, Shi D, Zhang C, Lin X, Liu Q, Dou R, Xiong B (2019). Crosstalk between cancer cells and tumor associated macrophages is required for mesenchymal circulating tumor cell-mediated colorectal cancer metastasis. Mol Cancer.

[43] Li L, Feng Q, Wang X (2020). PreMSIm: an R package for predicting microsatellite instability from the expression profiling of a gene panel in cancer. Comput Struct Biotechnol J.

[44] Chen B, Khodadoust MS, Liu CL, Newman AM, Alizadeh AA (2018). Profiling tumor infiltrating immune cells with CIBERSORT. Methods Mol Biol.

