# Supplementary figures and images for: Screening and identification of immune-related genes for immunotherapy and prognostic assessment in colorectal cancer patients
Source: BMC Med Genomics. 2022 Aug 8;15:177. doi: 10.1186/s12920-022-01329-2 (PMC9358808; doi:10.1186/s12920-022-01329-2)

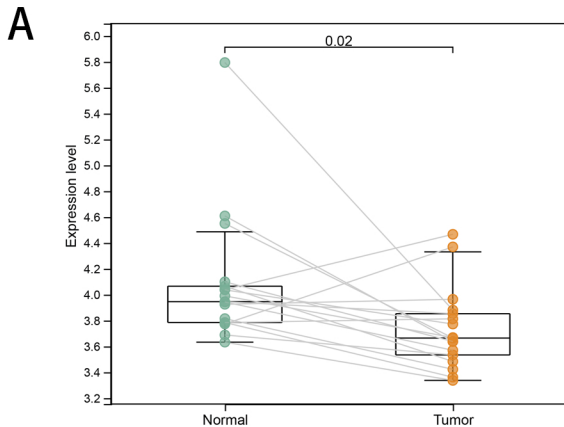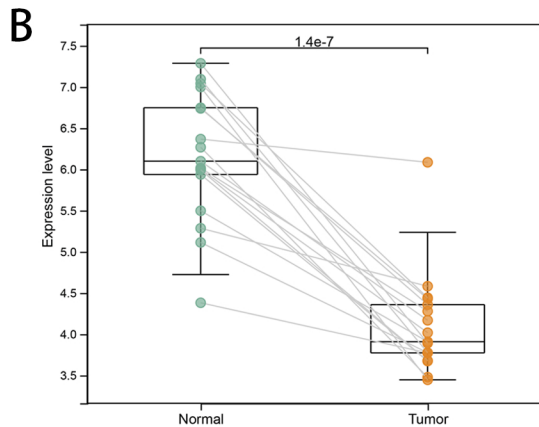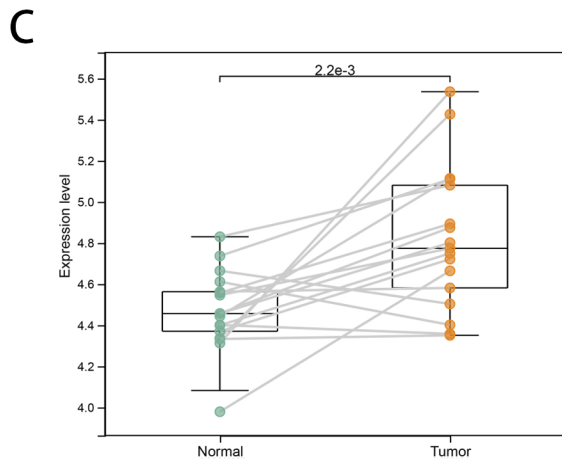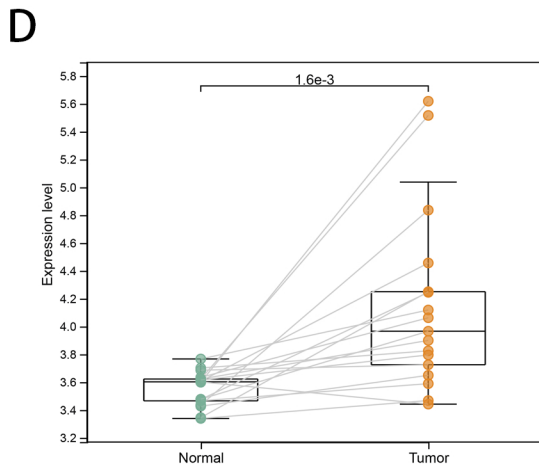

Supplement: Supplementary file 1 — Additional file 1. Fig. S1: The box diagram of expression of prognostic characteristic IRGs. From A to D, the expression box diagram of FGF2, SCG2, POMC, and TNFRSF19 is shown in the figure. Green is the paracancer tissue, yellow is the cancer tissue, and the line in the middle indicates that they belong to the same sample. [file 12920_2022_1329_MOESM1_ESM.pdf]

**A**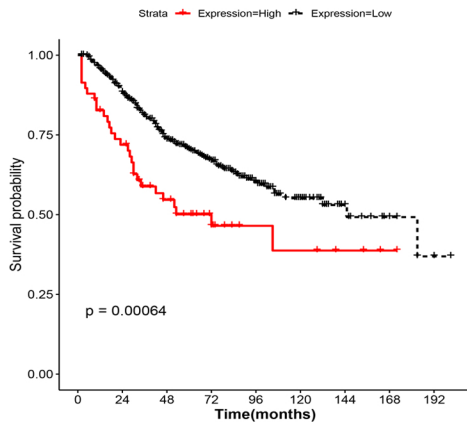**B**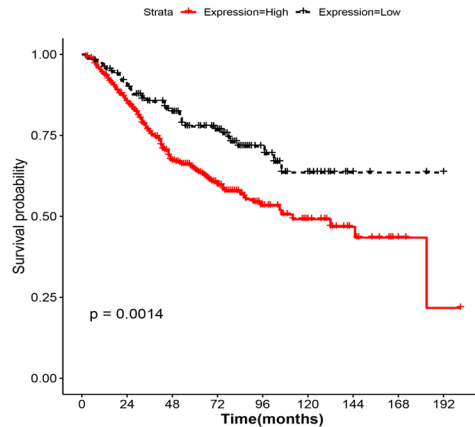**C**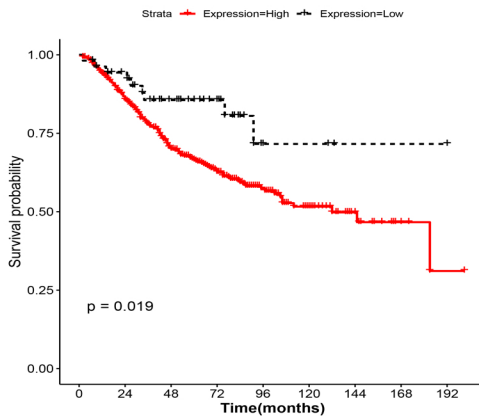**D**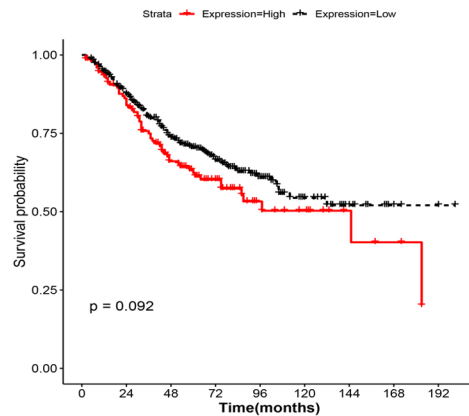

Supplement: Supplementary file 2 — Additional file 2. Fig. S2: Correlation between IRGs and prognosis from GEO database. The survival curves of FGF2, SCG2, POMC, and TNFRSF19 were shown from A to D. In the figure, red represents high expression group and black represents low expression group. [file 12920_2022_1329_MOESM2_ESM.pdf]

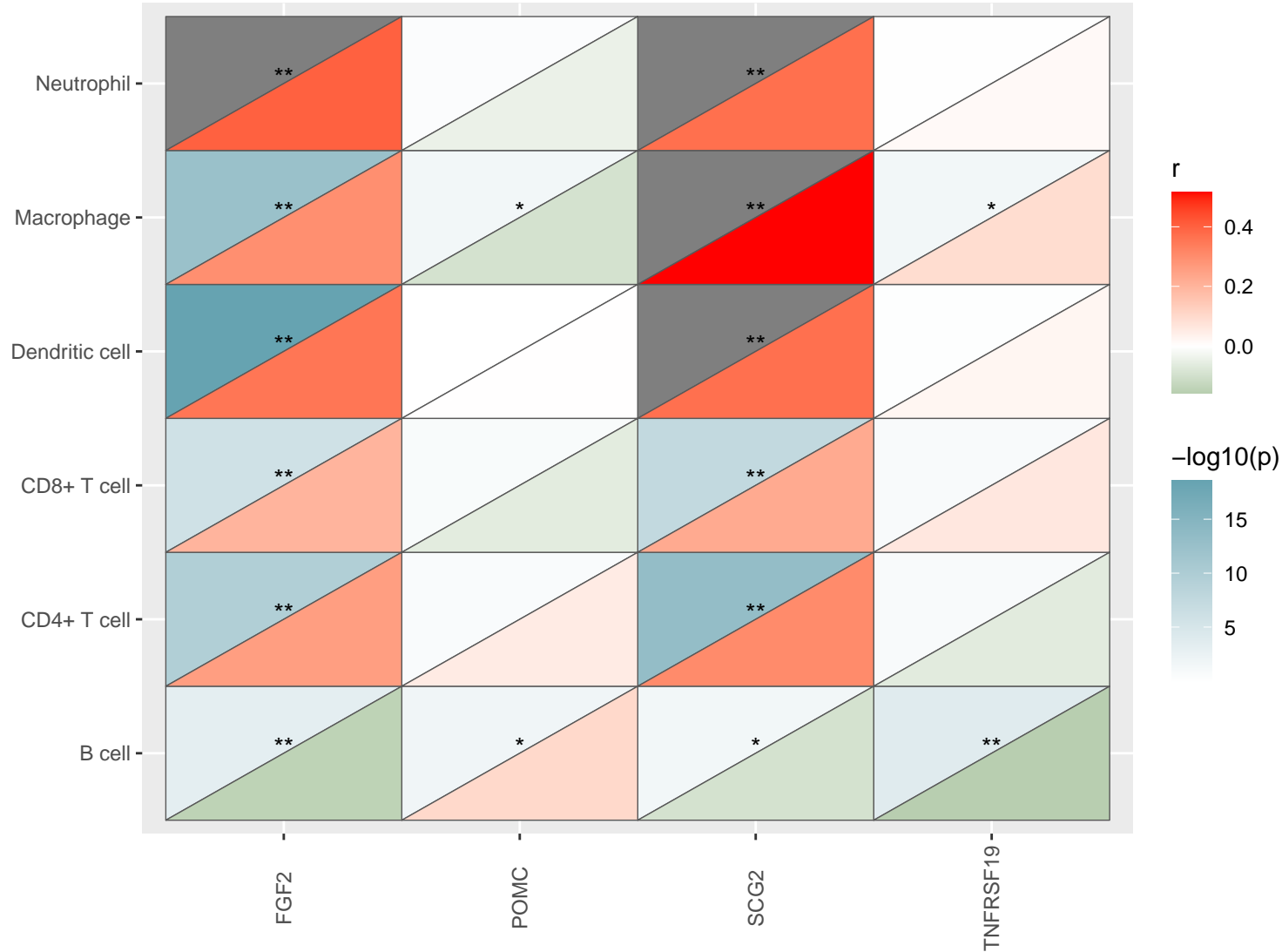

Supplement: Supplementary file 3 — Additional file 3. Fig. S3: Validation of immunocorrelation of IRGs from GEO database. From left to right are heat maps of correlations between FGF2, POMC, SCG2, TNFRSF19 and immune cells. The top left corner of each small square in the figure represents significance, and * represents p < 0.05, ** represents p < 0.01. The lower right corner shows correlation, green to red shows significance from negative to positive, and the deeper the correlation coefficient is, the greater the absolute value. [file 12920_2022_1329_MOESM3_ESM.pdf]

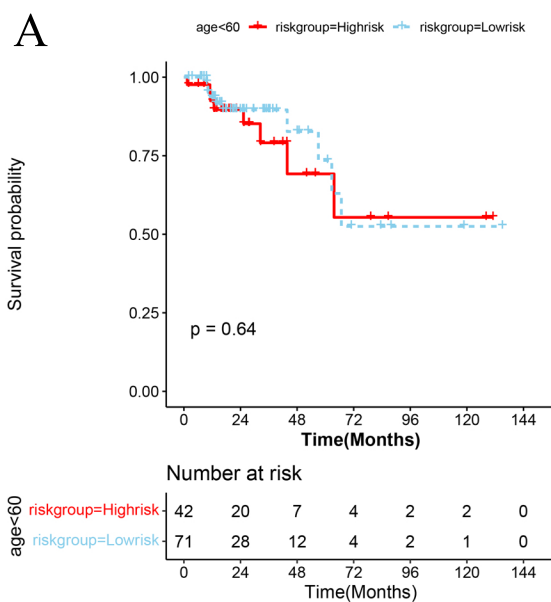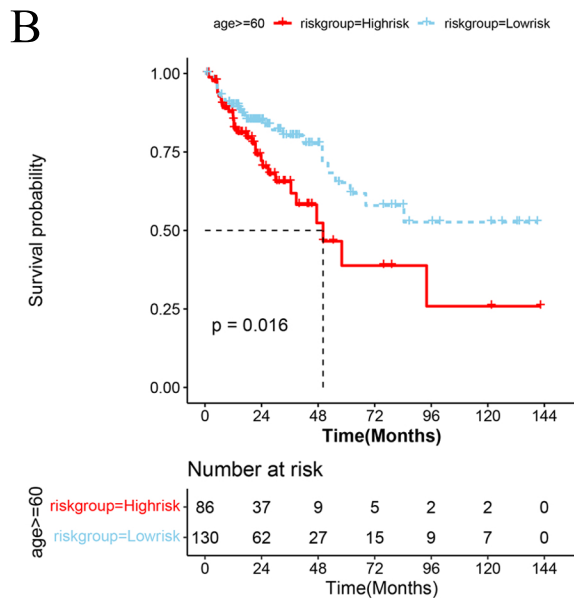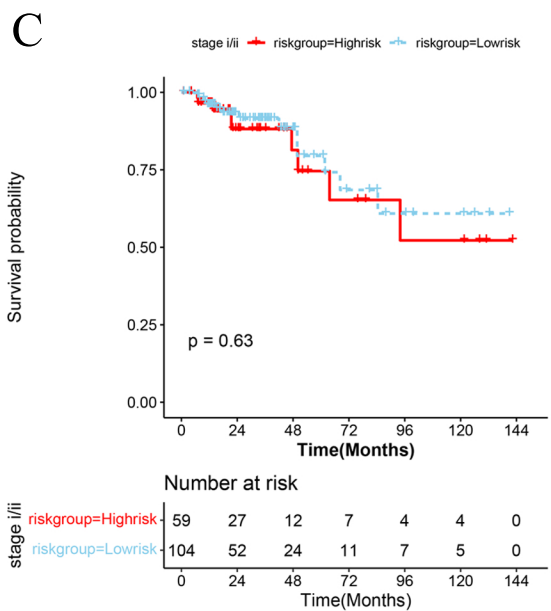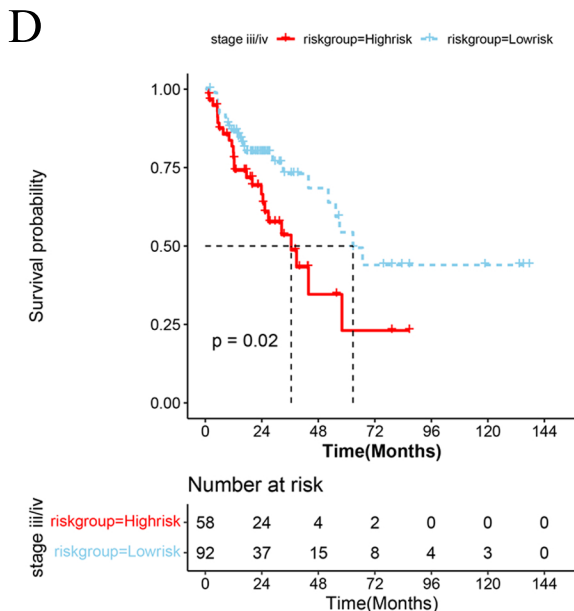

Supplement: Supplementary file 4 — Additional file 4. Fig. S4: The K-M survival curve of survival analysis adjusting for age and tumor stage. (A) K-M survival curve of patients under 60 years in the high-low risk group. (B) K-M survival curve of patients over 60 years in the high-low risk group. (C) K-M survival curve of patients in stage I-II. (D) K-M survival curve of patients in stage III-IV. [file 12920_2022_1329_MOESM4_ESM.pdf]

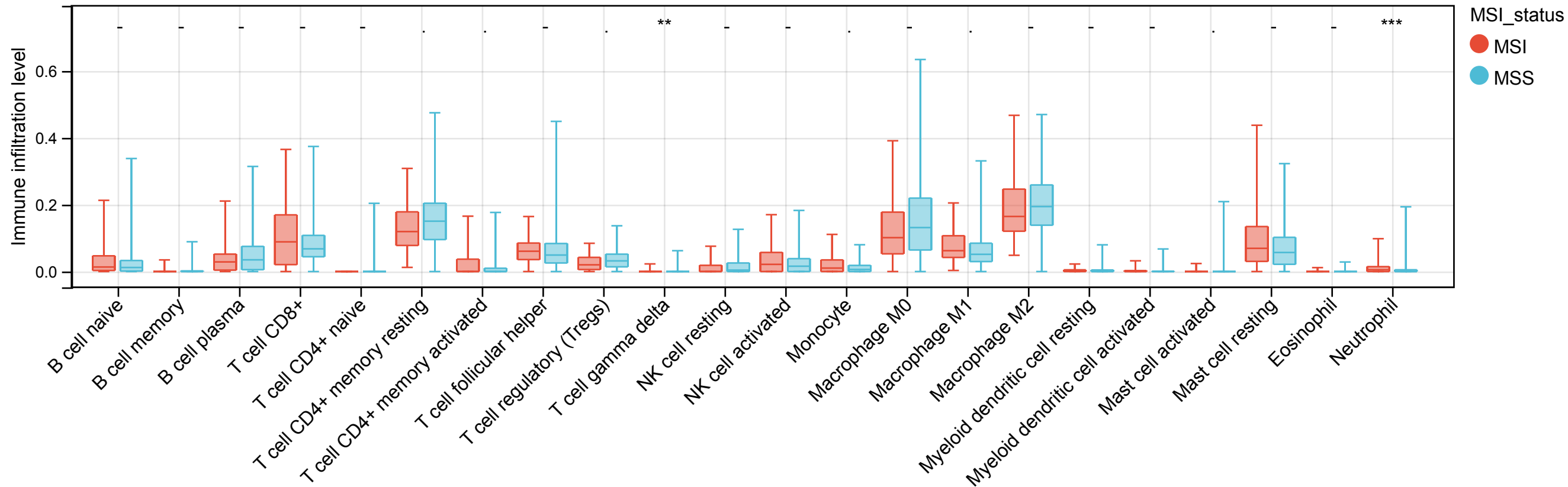

Supplement: Supplementary file 5 — Additional file 5. Fig. S5: The levels of immune cells infiltration in MSI and MSS groups. T cell Gamma Delta and Neutrophi showed a significant difference in the level of cell infiltration (p < 0.05), while the other cells showed no significant difference in infiltration between the two groups (p > 0.05). [file 12920_2022_1329_MOESM5_ESM.pdf]
